# Apriori Algorithm for the Data Mining of Global Cyberspace Security Issues for Human Participatory Based on Association Rules

**DOI:** 10.3389/fpsyg.2020.582480

**Published:** 2021-02-09

**Authors:** Zhi Li, Xuyu Li, Runhua Tang, Lin Zhang

**Affiliations:** ^1^ School of Media and Law, NingboTech University, Ningbo, China; ^2^ College of Media and International Culture, Zhejiang University, Hangzhou, China; ^3^ School of Computer and Data Engineering, NingboTech University, Ningbo, China; ^4^ School of Journalism and Communication, Dalian University of Foreign Languages, Dalian, China; ^5^ Faculty of Humanities and Arts, Macau University of Science and Technology, Taipa, Macau

**Keywords:** association rules, data mining, network sovereignty, cyberspace security, Apriori algorithm

## Abstract

This study explored the global cyberspace security issues, with the purpose of breaking the stereotype of people’s cognition of cyberspace problems, which reflects the relationship between interdependence and association. Based on the Apriori algorithm in association rules, a total of 181 strong rules were mined from 40 target websites and 56,096 web pages were associated with global cyberspace security. Moreover, this study analyzed support, confidence, promotion, leverage, and reliability to achieve comprehensive coverage of data. A total of 15,661 sites mentioned cyberspace security-related words from the total sample of 22,493 professional websites, accounting for 69.6%, while only 735 sites mentioned cyberspace security-related words from the total sample of 33,603 non-professional sites, accounting for 2%. Due to restrictions of language, the number of samples of target professional websites and non-target websites is limited. Meanwhile, the number of selections of strong rules is not satisfactory. Nowadays, the cores of global cyberspace security issues include internet sovereignty, cyberspace security, cyber attack, cyber crime, data leakage, and data protection.

## Introduction

Association rules, reflecting the interdependence and correlation between one thing and others, are one of the critical research methods in the data mining of graphic patterns (Epifania et al., 2020). In other words, an association rule is a practical and straightforward knowledge model implied in the data through quantified numbers, which mines the correlation relationship among valuable data items from massive data (Smink et al., 2019). At present, the data mining technology of association rules is mostly based on the Apriori algorithm, of which the core optimization lies in finding all the frequent item-sets in the transaction database (Shashi et al., 2020). Data mining is an essential branch of artificial intelligence. Celik (2019) expounded another view of the Apriori algorithm for data mining of association rules, finding hidden information processes through an algorithm in massive data information (Celik, 2019).

Regarding the mining issues of association rules, Agrawal and Elabbadi (1994) first proposed the Apriori algorithm, and discovered the potential association relationship among different items in a customer transaction database in 1995 (Li et al., 2005). Most algorithms are association rules discovered based on the massive data. The Apriori algorithm is mainly divided into two steps. The first step is to use the zhega universal transaction database to find all item-sets that satisfy the minimum confidence threshold. The second step is to find all the item-sets whose support degree are greater than the threshold value and to find the strong association rules with the confidence greater than the threshold (Hiba et al., 2020). However, in the process of the Apriori algorithm, this study used the inverse monotonicity of support and confidence of item-sets. In other words, the support and confidence of replaced item-sets are not higher than that of the original. This characteristic of the Apriori algorithm can be used to remove frequent item-sets, thus reducing computational load. There is a fatal flaw in the Apriori algorithm. During mining association rules, the transaction database should be traversed repeatedly to mine the time-consuming growth index with the increasing data volume (Yan et al., 2019). After development, the Apriori algorithm has established a closed item-set theory, widely used in medicine, finance, Internet, and other fields.

Psychology was used to confirm the five personality traits, meta traits, and the hypothetical relationship between self-esteem and the legal network terminology network (Rogoza et al., 2018). Although the social information processing theory integrates the literature on humility and resilience, it fails to explore its contextual triggers (Zhu et al., 2019). In terms of social exchange theory, the study focuses on the role of social interaction, mining samples to test hypothetical models on data (Qian et al., 2018). Besides, the social business model illustrates that information and communication technology can be integrated with transportation service providers and government resources (Wu et al., 2020). As a popular means of obtaining information, social media can mine bilingual texts, and promote the exploration of topics and market trends, gaining essential insights from crowd intelligence (Shen et al., 2019).

Association-rule mining includes mining frequent item-sets and discovering strong association rules (Jongseong et al., 2020). Mining frequent item-sets is an important step, and the Apriori class proposes many algorithms for mining frequent item-sets (Mary et al., 2016). The so-called frequent item-set mining is one of the steps of association rule mining, and the association rule is a close association between the item-sets that frequently appear on a given training item-set and the others (Sharmila and Vijayarani, 2020). In the process of scanning the data set, the Apriori algorithm uses an automatic recursive connection to mine the candidate item-sets (Hossain et al., 2020). Then pruning is used to mine frequent item-sets. The Apriori algorithm can mine all item-sets of large data sets (Huang, 2012). However, the data set is scanned repeatedly in order to ensure accuracy, resulting in a large number of candidate item-sets.

When association rules are mined from frequent item-sets, the method of using the “support-confidence” model has been recognized by most researchers. In recent years, Hazarika and Rahman (2014) found that mining association rules with the “support-confidence” model make it easy to produce the significance of the research conclusion. Meanwhile, the procedure is convenient to operate (Hazarika and Rahman, 2014). Brin et al. (1997) and his group first proposed the concept of interest and used the lift metric and chi-square test to mine relevant rules, which overcomes the shortcomings of “support-confidence” model (Brin et al., 1997).

Based on the association rules of the Apriori algorithm in data mining, global cyberspace security was studied, to seek the focus of current cyberspace issues and to provide a path reference for future cyberspace governance (Johns, 2019). The process can be divided into three stages. The first stage determines the relevant cyberspace security lexicon and selects target websites on the global Internet (Lintern, 2018). In the second stage, Python is used as a crawler tool to obtain the news hyperlink of target websites, and then the news page is segmented according to the lexicon mentioned in the first stage. Based on the word segmentation results in the second stage, the Apriori algorithm for data mining of association rules is analyzed in the third stage.

## Lexicon Selection of Target Websites in Global Cyberspace Security

It is essential to select appropriate target websites, thus ensuring the validity of the keywords database. Meanwhile, this study focused on target websites of global cyberspace security, which were divided into two types, professional and non-professional, to consider the comprehensiveness of data coverage.

Professional websites include internet sovereignty, data breach, cyber attacks, and rogue software, involving multiple aspects of cyberspace security. However, non-professional websites are based on the information content published by mainstream news media. Although the content is relatively small compared to professional websites, it involves rich information about global cyberspace security.

In the implementation of the first stage, professional websites and non-professional news websites were selected explicitly in global cyberspace security as the databases for lexicon selection. The ratio of the selected lexicon is generally maintained in a range of 1:1. A total of 15 target professional websites (See Table 1) and 25 non-professional target news websites were selected (See Table 2). The two types of target websites covered China, the United States, the United Kingdom, Germany, France, India, and other Internet developed and developing countries.

**Table 1:** Target professional websites.

No.	Name of institution	Website address
1	Info Security Magazine	http://www.infosecurity-magazine.com
2	The First Stop For Security News	https://threatpost.com/
3	The Hacker News	https://thehackernews.com/
4	National Security Agency	https://www.nsa.gov/
5	European Cyber And Information Security Agency	https://www.enisa.europa.eu/
6	Internet Governance Forum	http://www.intgovforum.org/multilingual/
7	European Telecommunications Standards Institute	https://www.etsi.org/
8	International Organization For Standardization	https://www.iso.org/conformity-assessment.html
9	International Telecommunication Union	https://www.itu.int/zh/itu-t/about/groups/pages/sg13.aspx
10	National Cyber Security Center (United Kingdom)	https://www.ncsc.gov.uk/
11	Australia Cyber Security Center	https://www.cyber.gov.au/
12	Airbus Cyber	https://airbus-cyber-security.com/
13	World Internet Conference	https://www.wicwuzhen.cn/
14	Cyberspace Administration Of China	http://www.cac.gov.cn/
15	Global Cyberspace Governance	https://www.pishu.com.cn/skwx_ps/sublibrary/14/10755.html

**Table 2:** Non-professional news target websites.

No.	Country	Media name	Website address
1	The US	The Huffington Post	http://www.huffingtonpost.com/
2		CNN	https://edition.cnn.com/
3		New York Times	https://www.nytimes.com/
4		Buzzfeed	https://www.buzzfeed.com/
5		Aol	https://www.aol.com/
6	The UK	BBC	http://www.bbc.co.uk/
7		Daily Mail	http://www.dailymail.co.uk/
8		The Guardian	https://www.theguardian.com.au/
9	France	Le Figaro	https://www.lefigaro.fr/
10		Le Parisien	http://www.leparisien.fr/
11		Reuters	http://reuters.com/
12		Agence France-Presse	https://www.afp.com/
13	Germany	Daily Mirror	https://www.tagesspiegel.de/
14		Le Monde	https://www.welt.de/
15	Russia	Rt Web Team	https://www.rt.com
16		ITAR-TASS	https://tass.ru/
17	Spain	Elmundo Es	https://www.elmundo.es/
18		Eleconomista Es	https://www.eleconomista.es/
19	China	CCTV	http://www.cctv.com/
20	Canada	The Globe And Mail	http://www.theglobeandmail.com/
21	Japan	Asahi Shimbun	http://www.asahi.com/ajw/?iref=comtop_usnavi
22	Singapore	zaobao	http://www.zaobao.com/
23		The New Pape	http://www.tnp.sg/
24	India	the Daily Telegraph	http://www.telegraph.co.uk/

The process of the first stage requires collecting lexicons to determine the necessary ones for the second stage of Python in the above website data crawler. As the basis for word segmentation, if the lexicons are not correctly selected, it is easy to miss critical data mining in the later analysis. This study selected 89 lexicons related to cyberspace security to cover all aspects of global cyberspace security, thus providing high-quality data for later association rules mining. The selected lexicons are listed as follows:

Cyberspace governance, cyberspace security, system security, information dissemination security, information content safety, internet ecosystem, cyber infrastructure security, application security (application system security), Internet security, Internet of things security, transaction security, database security, mobile security, risk management, risk assessment, information disclosure, communication technology, cyber technology, cyber protocol security, cyber running security, local area cyber (cyber security inspection), computer viruses, information alterations (loss), media security, and environmental security, equipment security, cyber security inspection, communication cyber, global governance, artificial intelligence, security strategy, cyber attacks, cyber security vulnerability, cyber law, information security, cyber threat, strategic proposition, cyber supervision, coordination mechanism, emergency management, social cyber, monitoring and early warning, cyber risk, cyber crime, security specification, security prevention, data protection, business secret, cosmopolitan web, data service, security framework, data breach, security event, security threat, cyber intrusion, and cyber security crisis, international cooperation, security system, information technology, encryption technology, cyber pattern, ICANN, cyber competitiveness, cyber deterrence theory, cyber self-management, Internet governance, cyberspace sovereignty, national safety, information infrastructure, cyber-culture, cyber terror, cyber governance, fundamentals of cyber security, cyberspace protection, international cyber cooperation, personal information protection, critical information infrastructure, multi-stakeholders, digital economic cooperation, personal privacy protection, digital divide and poverty, international internet system, cyber ecological governance, internet governance, cyber rules, cyber laws, cyber sovereignty, and cyber monitoring.

## Data Mining and Apriori Algorithm for Association Rule Analysis

Association rules are similar to the implication expression of X ⟹ Y, where X and Y are disjoint subsets, that is, X∩Y = Ø (Guo et al., 2017). The strength of the expression can be measured with support and confidence. Support is to determine how often association rules can be used for a given dataset, while confidence determines how often Y occurs in the transactions that contain X. Strong rules satisfy both the minimum support threshold (Minsup) and minimum confidence threshold (Minconf) rules (Watkins et al., 2020). Moreover, support is an important measure (Johnston and Baker, 2020). Since the rule with low support may emerge by chance, it rarely occurs in the entire dataset. Therefore, support is usually used to delete meaningless rules. Besides, it has the desired nature to discover association rules (Tightiz et al., 2020). However, confidence is inferred through association rules.

For a given X ⟹ Y, the higher the confidence, the greater the probability that Y is included in the transaction of X. Certainly, confidence can also estimate the conditional probability of Y at a given X (Sharadqah and Mojirsheibani, 2020). For example, cyber attacks ⟹ data breach, the higher the support, the higher the frequency of data breach and cyber attacks occur in a given dataset. Meanwhile, the higher the confidence, the higher the probability of data breach after cyber attacks.

Apriori algorithm of a mining association rule is based on two core theories: the subsets of frequent item-sets are frequent item-sets, and the supersets of infrequent item-sets are infrequent item-sets (Goldhammer et al., 2020). Frequent item-set refers to the set with several items that often appear, the support of which is greater than the minimum threshold (Minsup); non-frequent item-set refers to the item-set with a support lower than the threshold (Nguyen et al., 2017). If {cyber attacks, data breach} is a frequent item-set, {cyber attacks} and {data breach} must be frequent item-sets. If {cyber attacks, data breach} is an infrequent item-set, {cyber attacks, data breach, and artificial intelligence}, {cyber attacks, data breach, and cyber crime}, or {cyber attacks, data breach, cybercrime, and artificial intelligence}, and other supersets are infrequent item-sets.

However, the Apriori algorithm uses an Iterative Method (Shashi et al., 2020). First, the candidate 1-item-set and the corresponding support are searched to obtain the frequent 1-item-set by pruning out the 1-item-set with lower support. Then the remaining frequent 1-item-set is connected to get the candidate frequent 2-item-set. Meanwhile, the real frequent 2-item-set is obtained through filtering out the candidate frequent 2-item-set with lower support. Using this iterative method to operate until the frequent k + 1 item-set cannot be found, the corresponding frequent k-item-set is the output of the algorithm (Surender and Hegde, 2020). For example, the data set D in this study has four records, namely, (1) cyber attack, data protection, and cybercrime; (2) data breach, data protection, and artificial intelligence; (3) cyber attack, data breach, data protection, and artificial intelligence; and (4) data breach and artificial intelligence.

The Apriori algorithm is used to find frequent k-item-sets, setting the minimum support to 50%. First, a candidate frequent 1-item-set is generated, including all five data and calculating the corresponding support. Secondly, pruning is performed after the calculation. Since the support of {cyber crime} 1-item-set is only 25%, it has to be cut off. Therefore, the final frequent 1-item-set, {cyber attacks, data breach, data protection, and artificial intelligence}, is linked to generate the candidate frequent 2-item-set, {cyber attacks, data breach), {cyber attacks, data protection}, {cyber attacks, artificial intelligence}, {data breach, data protection}, {data breach, artificial intelligence}, and {data protection, artificial intelligence}, with a total of six groups. The first round of iteration ends at this point.

In the second round of iteration, the scanned data set is used to calculate the support of the candidate frequent 2-item-set, and then the item-sets are removed (Shariq, 2020). The support of {cyber attacks, data breach} and {cyber attacks, artificial intelligence} is only 25%, thus the frequent 1-item set is screened out to generate the real frequent 2-item set, {cyber attacks, data protection}, {data breach, data protection}, {data breach, artificial intelligence}, and {data protection, artificial intelligence}. Next, the four groups of frequent 2-item-sets are linked to generate a candidate frequent 3-item-set, {cyber attack, data breach, and data protection}, {cyber attacks, data protection, and artificial intelligence}, and {data breach, data protection, and artificial intelligence}. Through the calculation of the support of the candidate frequent 3-item-set, the support of {cyber attacks, data breach, data protection} and {cyber attacks, data protection, artificial intelligence} are both 25%. Therefore, the data needs to be pruned again to obtain the real frequent 3-item-set {data breach, data protection, and artificial intelligence}. Because there is only one frequent item-set remaining, no more data is linking at this stage. The candidate frequent 4-item-set is obtained, the final result of which is the frequent 3-item-set {data breach, data protection, and artificial intelligence}.

For the frequent item-set of {data breach, data protection, and artificial intelligence}, the subsets are {data breach}, {data protection}, {artificial intelligence}, {data breach, data protection}, {data breach, artificial intelligence}, and {data protection, artificial intelligence}. The rules are as follows:

Data Breach ⟹ Artificial Intelligence^Data Protection.
Data Protection ⟹ Data Breach^Artificial Intelligence.
Artificial Intelligence ⟹ Data Breach^Data Protection.
Data Breach^Data Protection ⟹ Artificial Intelligence.
Data Breach^Artificial Intelligence ⟹ Data Protection.
Data Protection^Artificial Intelligence ⟹ Data Breach.

Therefore, based on the data mining of global professional and non-professional target websites, the Apriori algorithm is used to analyze association rules. Combining this association rule with a series of attributes can present the specific information content of cyberspace security on global professional and non-professional target websites, which marks the completion of mining association rules for transaction databases at the third stage.

## Specific Presentation of Global Cyberspace Security Issues

The specific presentation of global cyberspace security issues is based on the association rule of the Apriori algorithm. The confidence formula of conf (I1->I3^I2) = support (I1, I2, I3)/support (I1) is used to calculate the confidence of each rule. Then the minimum confidence and minimum support are compared to mine the strong rules corresponding to the data (Ahmed and Tien, 2016).

However, the value of the corresponding rule can be analyzed through a series of attributes of association rules. (1) Support representing the support of the union of the former and the latter items (Joki et al., 2020); (2) Confidence involves the rules to identify the rule support/rule leader (Lin and James, 2020); (3) Lift refers to the ratio of the probability of containing an left-hand side (LHS) and an right-hand side (RHS) to the probability of containing RHS (Musab et al., 2019). It reflects the correlation between the LHS and the RHS in association rules. When the lift is larger than one, the higher it is, the higher the positive correlation is; when the lift is lower than one, the lower it is, the higher the negative correlation is. Meanwhile, there is no correlation when the lift is equal to one. (4) Leverage indicates the number of times that the LHS and RHS appear together when they are independently distributed. When the leverage is equal to zero, the LHS and RHS are independent (Kaveh et al., 2020). The larger the leverage is, the closer the relationship between the LHS and RHS. (5) Conviction is used to measure the independence of the LHS and RHS. Similar to the lift, the greater the value of confidence is the greater, the correlation is the greater (Unvan, 2020).

After the statistic of word segmentation and word frequencies of professional and non-professional target websites involved in global cyberspace issues, the Apriori algorithm is used to mine association rules and set threshold based on word frequency results. As a result, a series of association rules greater than the minimum support, and minimum confidence is obtained. Meanwhile, the patterns of LHS and RHS of association rules show the relationship among different word frequencies (Dario and Solange, 2019). The rule of “data breach to cyber-attack” reveals the connection between the data breach and cyber attacks. In terms of probability theory, when a data breach occurs, there will be cyber attacks with the probability depending on the size of confidence. The greater confidence of rules means that there are more sufficient reasons to trust the rule (Komiya et al., 2020).

Meanwhile, the rule has an essential attribute of support, which indicates the frequency of rules occurring in this data set. The larger the threshold is, the more frequently the rule occurs (Reigal et al., 2020). If both the two thresholds are relatively large, the data breach is often accompanied by cyber attack. Table 3 shows the details.

**Table 3:** Strong rules above the threshold of global professional target websites.

No.	LHS	RHS	Support	Confidence	Lift	Leverage	Conviction
1	Cyberspace security, Cyberspace governance⟹	Cyber sovereignty	0.12732	0.96239	6.61387	0.10807	22.71992
2	Cyber sovereignty⟹	Cyberspace security	0.13512	0.92857	5.30847	0.10966	11.55108
3	Cyberspace governance⟹	Cyber sovereignty	0.13252	0.92763	6.37500	0.11173	11.80749
4	Information technology, Cyberspace governance⟹	Cyber sovereignty	0.04417	0.89675	6.16274	0.03701	8.27554
5	Cyber crimes, International cooperation⟹	Internet governance	0.01537	0.87147	7.36255	0.01328	6.85954
6	Artificial intelligence, Cyber sovereignty⟹	Cyberspace security	0.05197	0.86957	4.97114	0.04151	6.32559
7	Cyberspace security, Information technology⟹	Information security	0.04821	0.86251	2.75249	0.03069	4.99422
8	National security, Cyberspace governance⟹	Information security	0.03030	0.85358	2.72399	0.01917	4.68963
9	Personal information protection⟹	Information security	0.02178	0.85281	2.72154	0.01378	4.66513
10	Security threats, Cyber attacks⟹	Information security	0.02488	0.84428	2.69430	0.01564	4.40941
11	Cyber crime, Cyberspace security⟹	Information security	0.02659	0.83944	2.67887	0.01667	4.27659
12	National security, Information technology⟹	Cyberspace security	0.02565	0.83906	4.79675	0.02030	5.12660
13	Artificial intelligence, Cyberspace governance⟹	Information security	0.04683	0.83366	2.66042	0.02923	4.12798
14	Cyber governance⟹	Cyberspace governance	0.02416	0.81835	5.72846	0.01994	4.71870
15	International rulemaking⟹	Information technology	0.01128	0.80952	8.55296	0.00996	4.75310
16	International cooperation, Cyber attack⟹	Information technology	0.01056	0.80591	8.51475	0.00932	4.66453
17	Intent governance⟹	Cyberspace security	0.03096	0.80344	4.59314	0.02422	4.19766
18	Cyber governance⟹	Information security	0.02366	0.80150	2.55778	0.01441	3.45913
19	Information technology Cyberspace governance⟹	Information security	0.03909	0.79349	2.53223	0.02365	3.32499
20	Infrastructure security⟹	Information technology	0.02460	0.75042	2.39478	0.01433	2.75121
21	National security, Information technology⟹	Information security	0.02272	0.74322	2.37180	0.01314	2.67404
22	Cyberspace security, Internet governance⟹	Information technology	0.02836	0.70564	2.25187	0.01577	2.33266
23	Artificial intelligence, Information technology⟹	Cyberspace security	0.02764	0.69638	3.98107	0.02070	2.71746
24	Information infrastructure⟹	Cyberspace security	0.03179	0.67807	3.87638	0.02359	2.56288
25	Artificial intelligence, Information technology⟹	Cyberspace governance	0.02676	0.67409	4.71866	0.02109	2.63004
26	Information infrastructure⟹	Information security	0.03068	0.65448	2.08861	0.01599	1.98728
27	International cooperation⟹	Cyberspace security	0.03345	0.64845	3.70704	0.02442	2.34694
28	Artificial intelligence, Information technology⟹	Information security	0.02527	0.63649	2.03120	0.01283	1.88893
29	Artificial intelligence⟹	Cyberspace security	0.06026	0.62500	3.57301	0.04340	2.20021
30	Artificial intelligence⟹	Cyber sovereignty	0.05976	0.61984	4.25975	0.04573	2.24771
31	International cooperation⟹	Cyberspace governance	0.03146	0.60986	5.15234	0.02535	2.25979
32	Information technology⟹	Cyber sovereignty	0.05457	0.57652	3.96203	0.04079	2.01777

Thresholds set by Python have the minimum support of 0.01 and minimum confidence of 0.3. According to this threshold, 6,876,606 strong rules are mined to facilitate data observation following the rules with equal support, remaining 181 rules. Table 3 lists 32 strong rules, while the remaining 149 rules are not analyzed because of low importance and little research value.

Table 3 shows that 181 strong rules are mined in 22,493 web pages of 15 global professional target websites, of which 32 strong rules are analyzed. Each row lists a strong rule and the corresponding support, confidence, lift, leverage, and conviction, which are arranged from largest to smallest according to confidence. From a whole perspective to study the 32 strong rules, it has little impact on judging confidence because of the vast amount of data and small support, and the threshold is still credible. However, the overall small support indicates that the words related to cyberspace security are comprehensive in global professional target websites. Moreover, the information discussing cyberspace security is relatively comprehensive and detailed. However, the threshold of confidence means that when some or a specific cyberspace security term in global target professional websites is mentioned, another term related to cyberspace security will be mentioned with a higher probability. The strong rule with the highest confidence is “Cyberspace Security, Cyberspace Governance ⟹ Cyber Sovereignty,” with the confidence of about 96.2%, showing that after referring to cyber sovereignty, there is a higher possibility of mentioning cyberspace security.

Meanwhile, the lifts of LHS and RHS of 32 strong rules higher than the threshold are both greater than one, indicating the positive correlation of word frequency between the two items. “International rule-making ⟹ information technology” and “international cooperation and cyber attacks ⟹ Information technology” have the highest lifts, which are 8.55296 and 8.51475, respectively. Information technology is mentioned most frequently by international rule-making, international cooperation, and cyber attacks in the field of global cyberspace security. Moreover, the leverages of LHS and RHS of the 32 strong rules are both more than zero, showing that the word frequency cohesion between the two items is higher than expected. The strong rules with the closest relationship are “cyberspace governance ⟹ cyber sovereignty,” “cyber sovereignty ⟹ cyberspace security” and “cyberspace security and cyberspace governance ⟹ cyber sovereignty,” the values of which are 0.11173, 0.10966, and 0.10807, respectively. Results show that cyberspace governance, cyber sovereignty, and cyberspace security in global cyberspace security have been mentioned and concerned frequently. However, “cyberspace security and security governance ⟹ cyber sovereignty” has the highest conviction among the 32 strong rules, which is 22.71992, the same as the maximum value of lift. The independence of “cyberspace security and security governance” and “cyber sovereignty” is strong and closely related, which are mentioned almost simultaneously.

Table 4 shows that the TOP 10 rules with the highest support and the corresponding confidence can be mined through 32 strong rules in global professional websites. First, the word frequency of cyber sovereignty is accompanied by the frequency of cyberspace security, cyberspace governance, artificial intelligence, and information technology. Then, the word frequency of artificial intelligence appears, followed by cyberspace security, cyber sovereignty, information security, and cyberspace governance. Besides, the word frequency of information technology is mentioned in the wake of cyber sovereignty, cyberspace security, information security, and cyberspace governance. Finally, analyzing from the rule with the highest support, “cyber sovereignty ⟹ cyberspace security,” cyberspace security appears in the confidence possibility of 92.8% after cyber sovereignty occurs. Moreover, analyzing from the top 10 rules, Cyber sovereignty has become the most frequent item in global target professional websites.

**Table 4:** Top 10 rules with the highest support for global professional target websites.

Top	LHS	RHS	Support	Confidence
1	Cyber sovereignty⟹	Cyberspace security	0.13512	0.92857
2	Cyberspace governance	Cyber sovereignty	0.13252	0.92763
3	Cyberspace security, Cyberspace governance⟹	Cyber sovereignty	0.12732	0.96239
4	Artificial intelligence⟹	Cyberspace security	0.06026	0.62500
5	Artificial intelligence⟹	Cyber sovereignty	0.05976	0.61984
6	Information technology⟹	Cyber sovereignty	0.05457	0.57652
7	Artificial intelligence, Cyber sovereignty⟹	Cyberspace security	0.05197	0.86957
8	Cyberspace security, Information technology⟹	Information security	0.04821	0.86251
9	Artificial intelligence, Cyberspace governance⟹	Information security	0.04683	0.83366
10	Information technology, Cyberspace governance⟹	Cyber sovereignty	0.04417	0.89675

Table 5 shows that the Top 10 strong rules with the highest confidence and the corresponding support can be mined through the 32 strong rules above the threshold. Then, the conclusions can be drawn as follows. Firstly, “cyberspace security and cyberspace governance ⟹ cyber sovereignty” has the highest confidence among the global target professional websites. The word frequency of “cyber sovereignty” appears after “cyberspace security and cyberspace governance,” which has higher support among all strong rules. Also, cyber sovereignty appears frequently and has higher support, whether as LHS, “cyber sovereignty ⟹ cyberspace security,” or as RHS, “cyberspace security and cyberspace governance ⟹ cyber sovereignty,” “cyberspace governance ⟹ cyber sovereignty,” and “information technology and cyberspace governance ⟹ cyber sovereignty.”

**Table 5:** Top 10 rules with the highest confidence for global target professional websites.

Top	LHS	RHS	Confidence	Support
1	Cyberspace security, Cyberspace governance⟹	Cyber sovereignty	0.96239	0.12732
2	Cyber sovereignty⟹	Cyberspace security	0.92857	0.13512
3	Cyberspace governance⟹	Cyber sovereignty	0.92763	0.13252
4	Information technology, Cyberspace governance⟹	Cyber sovereignty	0.89675	0.04417
5	Cyber crime, International cooperation⟹	Internet governance	0.87147	0.01537
6	Artificial intelligence, Cyber sovereignty⟹	Cyberspace security	0.86957	0.05197
7	Cyberspace security, Information technology⟹	Information security	0.86251	0.04821
8	National security, Cyberspace governance⟹	Information security	0.85358	0.03030
9	Individual information protection⟹	Information security	0.85281	0.02178
10	Security threats, Cyber attacks⟹	Information security	0.84428	0.02488

When any word frequency of “cyber sovereignty” or “artificial intelligence and cyber sovereignty” appears, cyberspace security has a high probability of appearing. When the word frequencies of “cyberspace security and information technology,” “national security and cyberspace governance,” “personal information protection,” and “security threats and cyber attacks” appear as LHS, “information security” frequently appears as RHS. After the appearance of “cyberspace security and information technology,” “national security and cyberspace governance,” “personal information protection,” or “security threats and cyber attacks,” “information security” occurs, with the probabilities of 86.2, 85.3, 85.2, and 84.4%, respectively.

Therefore, after comparing the Top 10 rules with the highest confidence and Top 10 rules with the highest support of global target professional websites, this study can obtain the following conclusions.

Firstly, although “cybercrime and international cooperation ⟹ Internet governance” does not appear in the Top 10 rules of support, it has the fifth-highest confidence. As long as cybercrime and international cooperation are mentioned in global professional target websites, there is a high probability of “Internet governance” appearing. Moreover, “cyber sovereignty ⟹ cyberspace security” rank high in both the Top 10 support rules and the Top 10 confidence rules, which mean cyber sovereignty and cyberspace security are often mentioned by the international community in global professional target websites. Meanwhile, cyberspace sovereignty is mentioned as information about cyberspace security.

Table 6 shows that a total of 181 strong rules are mined in 33,603 web pages of 25 global non-professional target websites, of which 27 strong rules are analyzed. Each row lists a strong rule and corresponding support, confidence, lift, leverage, and conviction, which are arranged from largest to smallest according to support. Though the study of 27 strong rules is from a whole perspective, the volume of useful information about cyberspace security mentioned in non-professional websites is less than that of professional websites. However, the overall support is slightly higher than that of professional websites. Meanwhile, the support of some strong rules is relatively high, indicating that the words related to cyberspace security are relatively simple when used in global non-professional websites, and the information on cyberspace security is monotonous. For example, the strong rule with the highest support is “cyber attacks ⟹ artificial intelligence,” with the support of about 33.7%, showing that cyber attacks and artificial intelligence are discussed more frequently in non-professional websites. Moreover, the rule with the highest confidence is “cyber sovereignty ⟹ Internet governance” in non-professional websites, with the confidence of about 92.8% and support of about 13.6%, showing that cyber sovereignty and Internet governance are discussed frequently in non-professional websites. Meanwhile, cyber sovereignty and Internet governance are mentioned with a high probability.

**Table 6:** Strong rules for global non-professional target websites above the threshold.

No.	LHS	RHS	Support	Confidence	Lift	Leverage	Conviction
1	Cyber attacks⟹	Artificial intelligence	0.33704	0.47867	1.41091	0.09816	1.26741
2	Cyber attacks⟹	Cyber crime	0.26585	0.37757	1.42022	0.07866	1.17948
3	Cyber crime⟹	Cyber crime, Artificial intelligence	0.14905	0.56067	1.66350	0.05945	1.50902
4	Cyber attacks, Artificial intelligence⟹	Cyber crime	0.14905	0.44224	1.66350	0.05945	1.31626
5	Cyber crime⟹	Artificial intelligence	0.14905	0.56067	1.65260	0.05886	1.50396
6	Cyber attacks, Cyber crime⟹	Artificial intelligence	0.14905	0.56067	1.65260	0.05886	1.50396
7	Artificial intelligence⟹	Cyber crime	0.14905	0.43934	1.65260	0.05886	1.30945
8	Artificial intelligence⟹	Cyber attacks, Cyber crime	0.14905	0.43934	1.65260	0.05886	1.30945
9	Cyber attacks⟹	Cyber crime, Artificial intelligence	0.14905	0.21169	1.42022	0.04410	1.07946
10	Cyber sovereignty⟹	Internet governance	0.13682	0.75000	5.43750	0.11166	3.44828
11	Cyber attacks⟹	Data breach	0.10790	0.15324	1.21913	0.01939	1.03253
12	Cyber attacks, Data breach⟹	Artificial intelligence	0.07453	0.69072	2.03593	0.03792	2.13637
13	Artificial intelligence⟹	Cyber attacks, Data breach	0.07453	0.21967	2.03593	0.03792	1.14324
14	Data breach⟹	Cyber attacks, Artificial intelligence	0.07453	0.59292	1.75919	0.03216	1.62857
15	Cyber attacks, Artificial intelligence⟹	Data breach	0.07453	0.22112	1.75919	0.03216	1.12252
16	Data breach⟹	Artificial intelligence	0.07453	0.59292	1.74766	0.03188	1.62311
17	Artificial intelligence⟹	Data breach	0.07453	0.21967	1.74766	0.03188	1.12043
18	Cyber attacks⟹	Data breach, Artificial intelligence	0.07453	0.10585	1.42022	0.02205	1.03503
19	Cyber attacks, Data breach⟹	Cyber crime	0.04894	0.45361	1.70625	0.02026	1.34363
20	Cyber crime⟹	Cyber attacks, Data breach	0.04894	0.18410	1.70625	0.02026	1.09340
21	Data breach⟹	Cyber crime	0.04894	0.38938	1.46466	0.01553	1.20230
22	Data breach⟹	Cyber attacks, Cyber crime	0.04894	0.38938	1.46466	0.01553	1.20230
23	Cyber attacks, Cyber crime⟹	Data breach	0.04894	0.18410	1.46466	0.01553	1.07158
24	Cyber crime⟹	Data breach	0.04894	0.18410	1.46466	0.01553	1.07158
25	Cyber attacks⟹	Data breach, Cyber crime	0.04894	0.06951	1.42022	0.01448	1.02210
26	Cyber attacks⟹	Data breach	0.04561	0.06477	1.38641	0.01271	1.01930
27	Cyber attacks⟹	Data breach, Cyber threat	0.03893	0.05529	1.42022	0.01152	1.01732

Thresholds set by Python have the minimum support of 0.01 and the minimum confidence of 0.05. According to this threshold, 181 strong rules are mined. Table 6 lists 27 strong rules, while the remaining 154 rules are not analyzed because of low importance and little research value.

The lifts of LHS and RHS of 27 strong rules higher than the threshold are both greater than 1, indicating the negative correlation of word frequency between the two items, and the relationship of mutual promotion is not significant. Moreover, the leverages of LHS and RHS of the 27 strong rules are both more than zero, showing that the word frequency cohesion between the two items is higher than expected. The strong rules with the closest relationship are “Cyber sovereignty ⟹ Internet governance,” “Cyber attacks ⟹ Artificial intelligence,” and “Cyber attacks ⟹ Cyber crime,” the values of which are 0.11166, 0.09816, and 0.07866, respectively. Results show that the international community generally pays attention to artificial intelligence, cyber sovereignty, cyber attacks, cyber crime, and Internet governance in the non-professional websites of global cyberspace security issues. “Cyber sovereignty ⟹ Internet governance” has the highest conviction among the 27 strong rules, which is 3.44828, while the maximum lift is the same as the maximum value of confidence. The independence of cyber sovereignty and Internet governance is strong and closely related, which are mentioned almost simultaneously.

Therefore, this study compared the strong rules of professional and non-professional websites as a whole. Firstly, cyber sovereignty is widely concerned by people. Among professional websites, the strong rule of “cyber sovereignty ⟹ cyberspace security” has the highest support, with the support of about 13.5%. In non-professional websites, “Internet sovereignty ⟹ Internet governance” is the tenth rule order by support, with the support of about 13.6%. Secondly, from the perspective of confidence, the word frequency of cyber sovereignty is usually accompanied by cyberspace security, cyberspace governance, artificial intelligence, and information technology in professional websites. Finally, based on confidence, the word frequency of cyber sovereignty is only accompanied by Internet governance in non-professional websites. For the same LHS, there are more diverse RHS in professional websites, but it is single in non-professional websites, showing that cyber sovereignty has been studied more comprehensively in professional websites.

Through analyzing the data mining accuracy in global professional and non-professional target websites, namely, the ratio of the number of websites with cyberspace security to the total number of websites, the following can be found.

**Figure 1:**
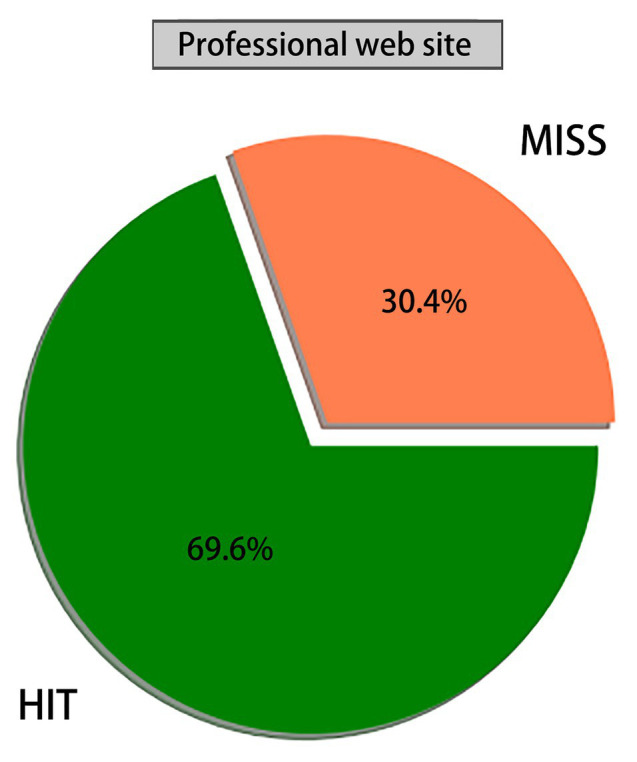
Proportion of websites containing cyberspace security in professional websites.

**Figure 2:**
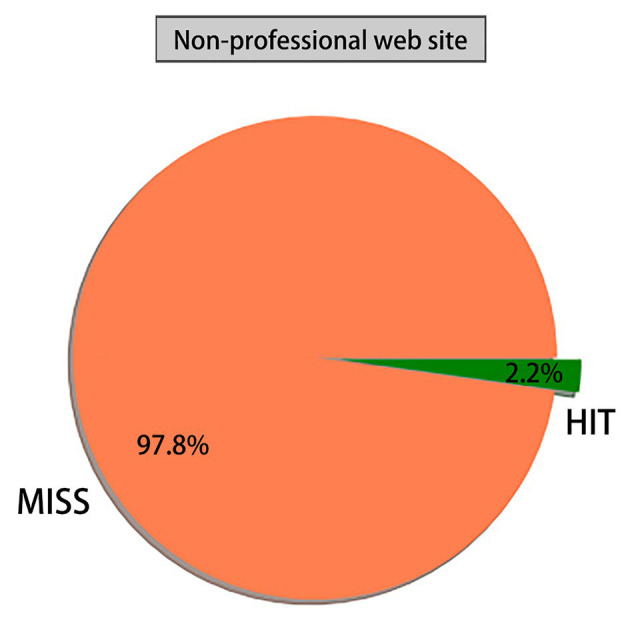
Proportion of websites containing cyberspace security in non-professional websites. MIS, Invalid information of MIS; HIT, Effective information of HIT.

1. Figure 1 shows that the number of websites containing cyberspace security in global professional target websites accounts for 69.6%, while that of websites without cyberspace security for 30.4%;
2. Figure 2 shows that the number of websites containing cyberspace security in global non-professional target websites accounts for 2%, while that of websites without cyberspace security for 97.8%. After further interpreting, 15,661 websites refer to cyberspace security from the total sample of 22,493 professional websites, accounting for 69.6%; meanwhile, 735 non-professional websites mentioned cyberspace security among the entire 33,603 sites, accounting for 2%. Therefore, there is a large gap in the proportion of valuable data between global professional and non-professional target websites. Meanwhile, the probability of cyberspace security appearing in professional target websites is far higher than that of non-professional websites, and the non-professional target websites pay far less attention to cyberspace security than professional websites.

**Figure 3:**
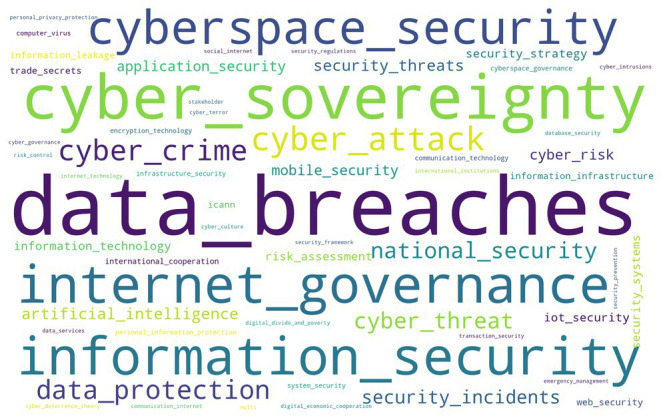
Word clouds of professional websites.

**Figure 4:**
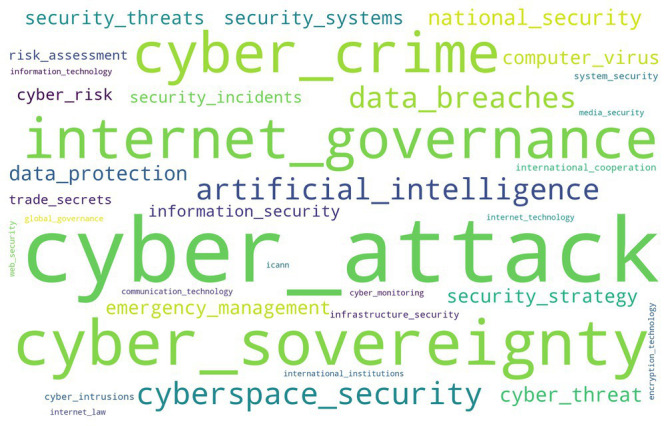
Word clouds of non-professional websites.

According to the statistics of word segmentation on the data mining of global target websites, the word frequency results can be generated into the word cloud of professional and non-professional target websites. If the word cloud appears more frequently in cyberspace, the larger the font size of the word, the more pronounced the problem will be. Figures 3, 4 show that the word cloud of professional target websites is richer than that of non-professional websites, and the information describing cyberspace security is comprehensive. Word frequencies of the data breach and cyber sovereignty in target professional websites, and cyber attacks and cyber sovereignty in non-professional websites are prominent, indicating that cyber security issues have been widely valued by the international professional field and mainstream news media.

Data breach, cyber sovereignty, Internet governance, information security, cyberspace security, national security, data protection, and cybercrime repeatedly appear in target professional websites, showing that the word frequencies involved in cyberspace security issues are professional and in-depth. However, the word frequencies of cyber sovereignty, cyber attacks, cybercrime, internet governance, security threat, computer virus, cyberspace security, cyber threat, artificial intelligence, and emergency management appear frequently in target non-professional websites. It shows that the mainstream news sites of the international community have only reported extensively on cyber security issues, lacking detailed and in-depth understanding.

Through comparing the word frequencies that often appear in target professional and non-professional websites, although the perspective of cyberspace security issues is different, the two types of websites focus on cyber sovereignty, cyber attacks, cyberspace security, Internet governance, national security, data breaches, and cyber threat.

Through sorting the word frequencies of global professional and non-professional target websites, the Top 10 strong rules of cyberspace security words with high frequency were obtained. If the proportion of word frequencies of the first 10 words is larger, the higher the ranking of the word is, the more frequently it is presented in global target websites, and the more it will be valued and recognized.

**Figure 5:**
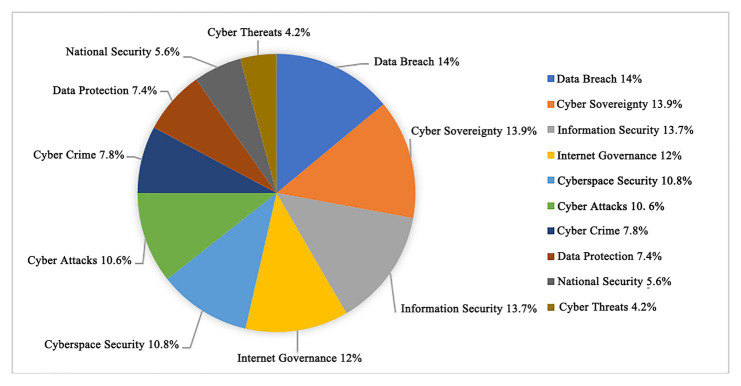
Proportion of Top 10 word frequencies of professional websites.

**Figure 6:**
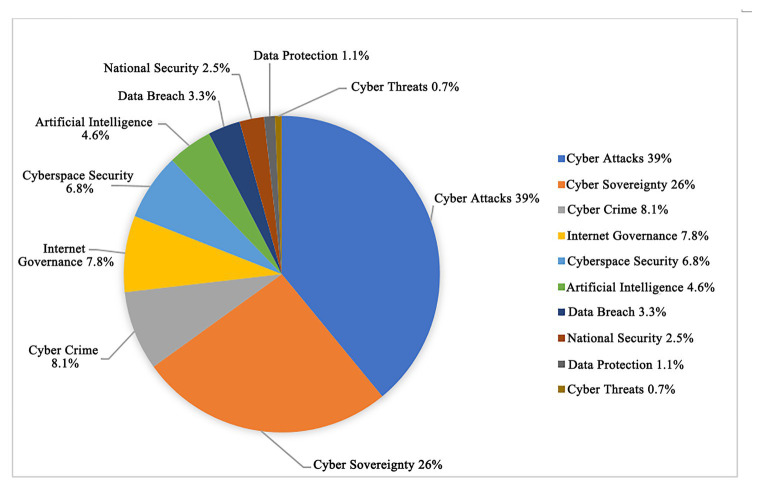
Proportion of Top 10 word frequencies of non-professional websites.

Figures 5, 6 show that the high word frequencies of Top 10 strong rules in professional websites are as follows: the word frequency of data leakage is 14%; that of cyber sovereignty 13.9%; that of information security 13.7%; that of Internet governance 12%; that of cyberspace security 10.8%; that of cyber attacks 10.6%; that of cyber crime 7.8%; that of data protection 7.4%; that of national security 5.6%; and that of the word cyber threat 4.2%. The high word frequencies of the Top 10 strong rules in non-professional websites are as follows: the word frequency of cyber attacks is 39%; that of cyber sovereignty 26%; that of Internet governance 8.1%; that of cyber crime 7.8%; that of cyberspace security 6.8%; that of artificial intelligence 4.6%; that of data leakage 3.3%; that of national security 2.5%; that of data protection 1.1%; and that of cyber threat 0.7%.

The frequent words of the Top 10 strong rules of global professional and non-professional target websites are compared to find that the frequent words of professional target websites are more evenly distributed. In contrast, those of the non-professional websites are unevenly distributed with cyber attacks in a dominant position.

Moreover, the word frequencies of global non-professional target websites are relatively broad, which are not as specific and accurate as that of professional websites. When presenting news related to cyberspace security, non-professional websites tend to use a single word frequency of cyber attacks. Conversely, professional websites use more specific and comprehensive words, adopting data breach, cyber sovereignty, information security, Internet governance, cyberspace security, and cyber attacks.

## Discussion

There are differences in cultural traditions and ideologies in different countries on the global Internet, which have pervaded the whole cyberspace. Moreover, cyberspace security issues have attracted worldwide attention. This study analyzed association rules based on the Apriori algorithm. Besides, the association rules were studied by selecting 15 professional target websites and 22,493 web pages, of which 15,661 websites are related to cyberspace security, accounting for 69.6% of the total target professional websites. A total of 25 non-professional target websites and 33,603 web pages are selected. Among them, 735 websites mention cyberspace security, accounting for 2% of the total number of non-professional websites. According to the threshold set by Python, the minimum support is 0.01, and the minimum confidence 0.05, with a total of 181 strong rules mined.

The 32 strong rules for the professional target website and 27 strong rules for the non-professional website are listed above. Moreover, the other strong rules are not analyzed due to their low importance and low research value. The two types of target websites cover China, America, Britain, Germany, France, India, and other developed and developing countries on the Internet. The results reflected the interdependence and correlation among global cyberspace security issues.

After the word clouds of global professional and non-professional target websites are compared, professional websites focus on the Top 10 high-frequency words containing a data breach, cyber sovereignty, information security, Internet governance, cyberspace security, cyber-attacks, cybercrime, data protection, national security, and cyber threat. Global non-professional websites, namely the mainstream news website of the international community, focus on the Top 10 high-frequency words of cyber attacks, cyber sovereignty, Internet governance, cybercrime, cyberspace security, artificial intelligence, data breach, national security, data protection, and cyber threats.

The limitation of the work lies in the limited number of data mining samples due to different language restrictions for global professional and non-target websites, which leads to an insufficient selection of strong rules. In future research, the following aspects will be completed: breaking through the language bottleneck, highlighting the number of selected data with strong rules, and taking mainstream countries as the target samples. A single country will be taken as a sample to analyze professional and non-professional websites involving cyber security issues. After concluding, the focus on cyber security issues between countries will be compared.

## Conclusion

Through comparing the word frequencies, the professional and non-professional target websites focused on cyber sovereignty, cyber attack, cyberspace security, Internet governance, national security, data leakage, and cyber threats. After sorting the word frequencies of global target professional websites and non-professional websites separately, the high word frequencies of the Top 10 strong rules of professional websites were data breach, cyber sovereignty, information security, Internet governance, cyberspace security, cyber attacks, cyber crime, data protection, national security, and cyber threats. Meanwhile, the high word frequencies of the Top 10 strong rules of non-professional websites were cyber attacks, cyber sovereignty, Internet governance, cyber crime, cyberspace security, artificial intelligence, data breach, national security, data protection, and cyber threats. Therefore, the focuses of current global cyberspace security issues were cyber sovereignty, Internet governance, cyberspace security, cyber attacks, cyber crime, national security, cyber threat, and data protection.

From an industry perspective, the first level of focus referred to data breach and cyber sovereignty. The second was Internet governance, information security, cyberspace security, architectural security, data protection, cyber crime, and cyber attacks. The third referred to cyber threat, artificial intelligence, application security, security threat, physical cyber security, and cyber risk. The fourth refers to security strategy, information technology, multi-stakeholder, Internet security, risk assessment, infrastructure security, and international cooperation.

In terms of the international community, the first level included cyber sovereignty and cyber attacks. The second included Internet governance, cybercrime, data protection, security threats, data breach, cyberspace security, emergency management, national security, security strategy, and security system. The third includes computer viruses, cyber risks, artificial intelligence, information security, international cooperation, ICANN, risk assessment, security incidents, trade secrets, and infrastructure security. The fourth includes Internet security, global governance, cyber monitoring, communication technology, cyber law, cyber technology, and system security.

Based on this level analysis, this study sorted out the security issues in global cyberspace, and summarized the specific security problems in cyberspace. It showed that the global cyberspace security issues were different from the identification of the international community. For the global governance of cyberspace security, effective and targeted governance solutions can be proposed according to this study, which is conducive to building a community with a shared future in cyberspace and constructing an Internet governance system.

## Data Availability Statement

The raw data supporting the conclusions of this article will be made available by the authors, without undue reservation.

## Ethics Statement

The studies involving human participants were reviewed and approved by Zhejiang University and Dalian University of Foreign Languages Ethics Committees. The patients/participants provided their written informed consent to participate in this study.

## Author Contributions

ZL designed the study, conceived the analysis question and conducted the analysis; XL and LZ conducted the analysis also and critically revised the manuscript content; and RT is the organizer of the project and responsible for sorting out the contact data and connecting with other researchers.

### Conflict of Interest

The authors declare that the research was conducted in the absence of any commercial or financial relationships that could be construed as a potential conflict of interest.

## References

[ref41] Agrawal D. Elabbadi A. (1994). A nonrestrictive concurrency control protocol for object oriented databases. Distrib. Parallel Dat. 2, 7–31.

[ref1] Ahmed S. Tien T. D. (2016). Bounded support and confidence over evidential databases. Procedia Comput. Sci. 80, 1822–1833. 10.1016/j.procs.2016.05.469

[ref2] Brin S. Motwani R. Silverstein C. (1997). Beyond market baskets: generalizing association rules to correlations. ACM Sigmod Rec. 26, 265–276. 10.1145/253262.253327

[ref3] Celik S. (2019). Comparing predictive performances of tree-based data mining algorithms and MARS algorithm in the prediction of live body weight from body traits. Pak. J. Zool. 51, 1447–1456. 10.17582/journal.pjz/2019.51.4.1447.1456

[ref4] Dario B. C. Solange O. R. (2019). Analysis of green manure decomposition parameters in Northeast Brazil using association rule cybers. Comput. Electron. Agric. 159, 34–41. 10.1016/j.compag.2019.02.013

[ref5] Epifania O. M. Anselmi P. Robusto E. (2020). DscoreApp: a shiny web application for the computation of the implicit association test d-score. Front. Psychol. 10:2938. 10.3389/fpsyg.2019.02938, PMID: 31998191 PMC6968522

[ref6] Goldhammer F. Scherer R. Greiff S. (2020). Editorial: advancements in technology-based assessment: emerging item formats, test designs, and data sources. Front. Psychol. 10:3047. 10.3389/fpsyg.2019.03047, PMID: 32038410 PMC6984335

[ref7] Guo Y. Wang M. X. Li X. (2017). Application of an improved apriori algorithm in a mobile e-commerce recommendation system. Ind. Manag. Data Syst. 117, 287–303. 10.1108/IMDS-03-2016-0094

[ref8] Hazarika M. Rahman M. (2014). Mapreduce based eclat algorithm for association rule mining in datamining. Int. J. Comput. Sci. Eng. 3, 19–18.

[ref9] Hiba B. Karima A. A. Youcef D. Jerry C. W. L. (2020). Data mining-based approach for ontology matching problem. Appl. Intell. 50, 1204–1221. 10.1007/s10489-019-01593-3

[ref10] Hossain T. M. Watada J. Jian Z. W. Sakai H. Rahman S. Aziz L. A. (2020). Missing well log data handling in complex lithology prediction: an nis apriori algorithm approach. Inter. J. Innov. Comput. I. 16, 1077–1091. 10.24507/ijicic.16.03.1077

[ref11] Huang T. C. K. (2012). Mining the change of customer behavior in fuzzy time-interval sequential patterns. Appl. Soft Comput. 12, 1068–1086. 10.1016/j.asoc.2011.11.017

[ref12] Johns B. T. (2019). Mining a crowdsourced dictionary to understand consistency and preference in word meanings. Front. Psychol. 10:268. 10.3389/fpsyg.2019.00268, PMID: 30833917 PMC6387934

[ref13] Johnston K. Baker J. (2020). Waste reduction strategies: factors affecting talent wastage and the efficacy of talent selection in sport. Front. Psychol. 10:2925. 10.3389/fpsyg.2019.02925, PMID: 31998188 PMC6967295

[ref14] Joki K. Bagirov A. M. Karmitsa N. Marko M. Sona T. (2020). Clusterwise support vector linear regression. Eur. J. Oper. Res. 287, 19–35. 10.1016/j.ejor.2020.04.032

[ref15] Jongseong K. Unil Y. Eunchul Y. Jerry C. W. L. Philippe F. V. (2020). One scan based high average-utility pattern mining in static and dynamic databases. Futur. Gener. Comput. Syst. 111, 143–158. 10.1016/j.future.2020.04.027

[ref16] Kaveh A. Bontis N. Zarina Z. (2020). The confluence of knowledge management and management control systems: a conceptual framework. Knowl. Process. Manag. 27, 133–142. 10.1002/kpm.1628

[ref17] Komiya A. Ozono H. Watabe M. Miyamoto Y. Ohtsubo Y. Oishi S. (2020). Socio-ecological hypothesis of reconciliation: cultural, individual, and situational variations in willingness to accept apology or compensation. Front. Psychol. 11:1761. 10.3389/fpsyg.2020.01761, PMID: 32793075 PMC7390922

[ref18] Li X. G. Jin R. M. Gagan A. (2005). Compiler and runtime support for shared memory parallelization of data mining algorithms. Int. Work. Lang. Compilers Par. Comput. 17, 71–89. 10.1007/11596110_18

[ref19] Lin C. J. James M. S. (2020). Efficient analysis of time-to-event endpoints when the event involves a continuous variable crossing a threshold. J. Stat. Plan. Inference 208, 119–129. 10.1016/j.jspi.2020.02.003, PMID: 32884165 PMC7097971

[ref20] Lintern G. (2018). Book review: cybercognition: brain, behaviour and the digital world. Front. Psychol. 9:1069. 10.3389/fpsyg.2018.01069

[ref21] Mary G. L. Karthi M. Rzvi T. (2016). A novel technique for multi-class ordinal regression-APDC. Indian J. Sci. Technol. 9, 1–8. 10.17485/ijst/2016/v9i10/88890

[ref22] Musab A. G. Laouamer L. Nana L. T. (2019). A blind spatial domain-based image watermarking using texture analysis and association rules mining. Multimed. Tools Appl. 78, 15705–15750. 10.1007/s11042-018-6851-2

[ref23] Nguyen L. T. Trinh T. Ngoc T. N. Vo B. (2017). A method for mining top-rank-k frequent closed item-sets. J. Intell. Fuzzy Syst. 32, 1297–1305. 10.3233/JIFS-169128

[ref24] Qian J. Song B. Jin Z. Wang B. Chen H. (2018). Linking empowering leadership to task performance, taking charge, and voice: the mediating role of feedback-seeking. Front. Psychol. 9:2025. 10.3389/fpsyg.2018.02025, PMID: 30410461 PMC6209672

[ref25] Reigal R. E. Hernandez M. A. Juarez R. M. R. Morales S. V. (2020). Physical exercise and fitness level are related to cognitive and psychosocial functioning in adolescents. Front. Psychol. 11:17777. 10.3389/fpsyg.2020.01777, PMID: 32793076 PMC7393645

[ref26] Rogoza R. Żemojtel P. M. Kwiatkowska M. M. Kwiatkowska K. (2018). The bright, the dark, and the blue face of narcissism: the spectrum of narcissism in its relations to the metatraits of personality, self-esteem, and the nomological network of shyness, loneliness, and empathy. Front. Psychol. 9:343. 10.3389/fpsyg.2018.00343, PMID: 29593627 PMC5861199

[ref27] Sharadqah A. A. Mojirsheibani M. (2020). A simple approach to construct confidence bands for a regression function with incomplete data. Adv. Stat. Anal. 10, 81–99. 10.1007/s10182-019-00351-7

[ref28] Shariq B. (2020). An efficient pattern growth approach for mining fault tolerant frequent item-sets. Expert Syst. Appl. 143:113046. 10.1016/j.eswa.2019.113046, PMID: 32288329 PMC7126664

[ref29] Sharmila S. Vijayarani S. (2020). Association rule mining using fuzzy logic and whale optimization algorithm. Soft Comput. 4, 18–34. 10.1007/s00500-020-05229-4

[ref30] Shashi R. Dharavath R. Krishan K. S. (2020). A spark-based apriori algorithm with reduced shuffle overhead. J. Supercomput. 77, 133–151. 10.1007/s11227-020-03253-7

[ref32] Shen C. W. Min C. Wang C. C. (2019). Analyzing the trend of O2O commerce by bilingual text mining on social media. Comput. Hum. Behav. 101, 474–483. 10.1016/j.chb.2018.09.031

[ref33] Smink W. A. C. Fox J. P. Sang E. T. K. Sools A. M. Westerhof G. J. Veldkamp B. P. (2019). Understanding therapeutic change process research through multilevel modeling and text mining. Front. Psychol. 10:1186. 10.3389/fpsyg.2019.01186, PMID: 31191394 PMC6548879

[ref34] Surender R. Hegde R. M. (2020). Optimal relay node selection in time-varying IoT cybers using apriori contact pattern information. Ad Hoc Netw. 98, 102–118. 10.1016/j.adhoc.2019.102065

[ref35] Tightiz L. Nasab M. A. Yang H. (2020). An intelligent system based on optimized ANFIS and association rules for power transformer fault diagnosis. ISA Trans. 103, 63–74. 10.1016/j.isatra.2020.03.022, PMID: 32197758

[ref36] Unvan Y. A. (2020). Market basket analysis with association rules. Commun. Stat. Theor. Methods 1, 1–14. 10.1080/03610926.2020.1716255

[ref37] Watkins C. D. Xiao D. K. Perrett D. I. (2020). Social transmission of leadership preference: knowledge of group membership and partisan media reporting moderates perceptions of leadership ability from facial cues to competence and dominance. Front. Psychol. 10:2996. 10.3389/fpsyg.2019.02996, PMID: 32010029 PMC6971406

[ref38] Wu Y. J. Liu W. J. Yuan C. H. (2020). A mobile-based barrier-free service transportation platform for people with disabilities. Comput. Hum. Behav. 107:105776. 10.1016/j.chb.2018.11.005

[ref39] Yan D. F. Zhao X. Lin R. H. Bai D. M. (2019). PPQAR: parallel PSO for quantitative association rule mining. Peer. Peer. Netw. Appl. 12, 1433–1444. 10.1007/s12083-018-0698-1

[ref40] Zhu Y. Zhang S. Shen Y. (2019). Humble leadership and employee resilience: exploring the mediating mechanism of work-related promotion focus and perceived insider identity. Front. Psychol. 10:673. 10.3389/fpsyg.2019.00673, PMID: 31001166 PMC6456677

